# COVID-19 related acute necrotizing encephalopathy with extremely high interleukin-6 and RANBP2 mutation in a patient with recently immunized inactivated virus vaccine and no pulmonary involvement

**DOI:** 10.1186/s12879-022-07610-0

**Published:** 2022-07-23

**Authors:** Thanakit Pongpitakmetha, Pasin Hemachudha, Wanakorn Rattanawong, Poosanu Thanapornsangsuth, Anand Viswanathan, Thiravat Hemachudha

**Affiliations:** 1 Department of Pharmacology, Faculty of Medicine, Chulalongkorn University, Bangkok, 10330 Thailand; 2 Chula Neuroscience Center, Faculty of Medicine, King Chulalongkorn Memorial Hospital, Chulalongkorn University, Bangkok, 10330 Thailand; 3 Thai Red Cross Emerging Infectious Diseases Health Science Centre, World Health Organization Collaborating Centre for Research and Training on Viral Zoonoses, King Chulalongkorn Memorial Hospital, Faculty of Medicine, Chulalongkorn University, Bangkok, 10330 Thailand; 4 Department of Medicine, Faculty of Medicine, King Mongkut’s Institute of Technology Ladkrabang, Bangkok, 10520 Thailand; 5 Division of Neurology, Department of Medicine, King Chulalongkorn Memorial Hospital, Faculty of Medicine, Chulalongkorn University, Bangkok, 10330 Thailand; 6 J. Philip Kistler Stroke Research Center, Department of Neurology, Massachusetts General Hospital, Harvard Medical School, Boston, MA USA

**Keywords:** COVID-19, Acute necrotizing encephalopathy, IL-6, *RANBP2*, COVID-19 vaccine

## Abstract

**Background:**

We report the first case of COVID-19 associated acute necrotizing encephalopathy (ANE) without pulmonary disease in a patient with an extremely high interleukin-6 (IL-6) level and Ran Binding Protein 2 (*RANBP2*) mutation.

**Case presentation:**

A 29-year-old woman recently immunized with inactivated viral vaccine—BBIBP32-CorV (Sinopharm) presented with alteration of consciousness. Her body temperature was 37° Celsius, blood pressure 42/31 mmHg, heart rate 130 bpm, respiratory rate 20 per minute, and oxygen saturation 98%. Respiratory examination was unremarkable. Neurological examination revealed stupor but preserved brainstem reflexes. Non-contrast computerized tomography of the brain showed symmetrical hypodense lesions involving bilateral thalami and cerebellar hemispheres characteristic of ANE. No pulmonary infiltration was found on chest radiograph. SARS-CoV-2 was detected by PCR; whole genome sequencing later confirmed the Delta variant. *RANBP2* gene analysis revealed heterozygous Thr585Met mutation. Serum IL-6 was 7390 pg/mL. Urine examination showed pyelonephritis. Her clinical course was complicated by seizure, septic shock, acute kidney injury, and acute hepatic failure. She later developed coma and passed away in 6 days.

**Conclusions:**

ANE is caused by cytokine storm leading to necrosis and hemorrhage of the brain. IL-6 was deemed as a prognostic factor and a potential treatment target of ANE in previous studies. *RANBP2* missense mutation strongly predisposes this condition by affecting mitochondrial function, viral entry, cytokine signaling, immune response, and blood–brain barrier maintenance. Also, inactivated vaccine has been reported to precipitate massive production of cytokines by antibody dependent enhancement (ADE). The true incidence of COVID-19 associated ANE is not known as were the predictors of its development. We proposed these potential two factors (*RANBP2* mutation and ADE) that could participate in the pathogenesis of ANE in COVID-19 apart from SARS-CoV2 infection by itself. Further study is needed to confirm this hypothesis, specifically in the post-vaccination period. Role of *RANBP2* mutation and its application in COVID-19 and ANE should be further elaborated.

## Background

After the emergence of severe acute respiratory syndrome coronavirus 2 (SARS-CoV-2) in 2019, millions of individuals have been infected worldwide. Upon those hospitalized, complications following the infection have been one of the major concerns. One of such, acute necrotizing encephalopathy (ANE) has been scarcely reported, however, the disease itself is devastating [1]. ANE has been previously reported in children and adults after an acute viral respiratory infection, mostly by influenza (H1N1 strain). The exact pathophysiology is unknown but several factors including specific gene mutations and cytokine productions from various causes prompt the evolution of this condition. *RANBP2* missense mutation strongly predisposes this condition by affecting mitochondrial function, viral entry, cytokine signaling, immune response, and blood–brain barrier maintenance [2]. The level of IL-6 is an indicator of a very poor prognosis in ANE in children [3].

Here, we report a recently immunized patient (BBIBP-CorV–inactivated vaccine) with coronavirus disease-2019 (COVID-19) and ANE, who had extremely high interleukin-6 (IL-6) level and Ran Binding Protein 2 (*RANBP2*) missense mutation (The first case in COVID-19).

## Case presentation

A 29-year-old woman without past medical history received the first dose of BBIBP-CorV (Sinopharm), whole-cell inactivated virus vaccine (IVV), in early July 2021 without immediate complication. Eight days later after the vaccination, she was found stuporous with fecal and urinary incontinence. Her initial vital signs showed body temperature of 37 °C, blood pressure 42/31 mmHg, heart rate 130 bpm, respiratory rate 20 per minute, and oxygen saturation 98%. Respiratory examination was unremarkable. Neurological examination revealed stupor (Glasgow coma scale was E2V1M4), but preserved brainstem reflexes and myoclonus at the left shoulder.

**Fig. 1:**
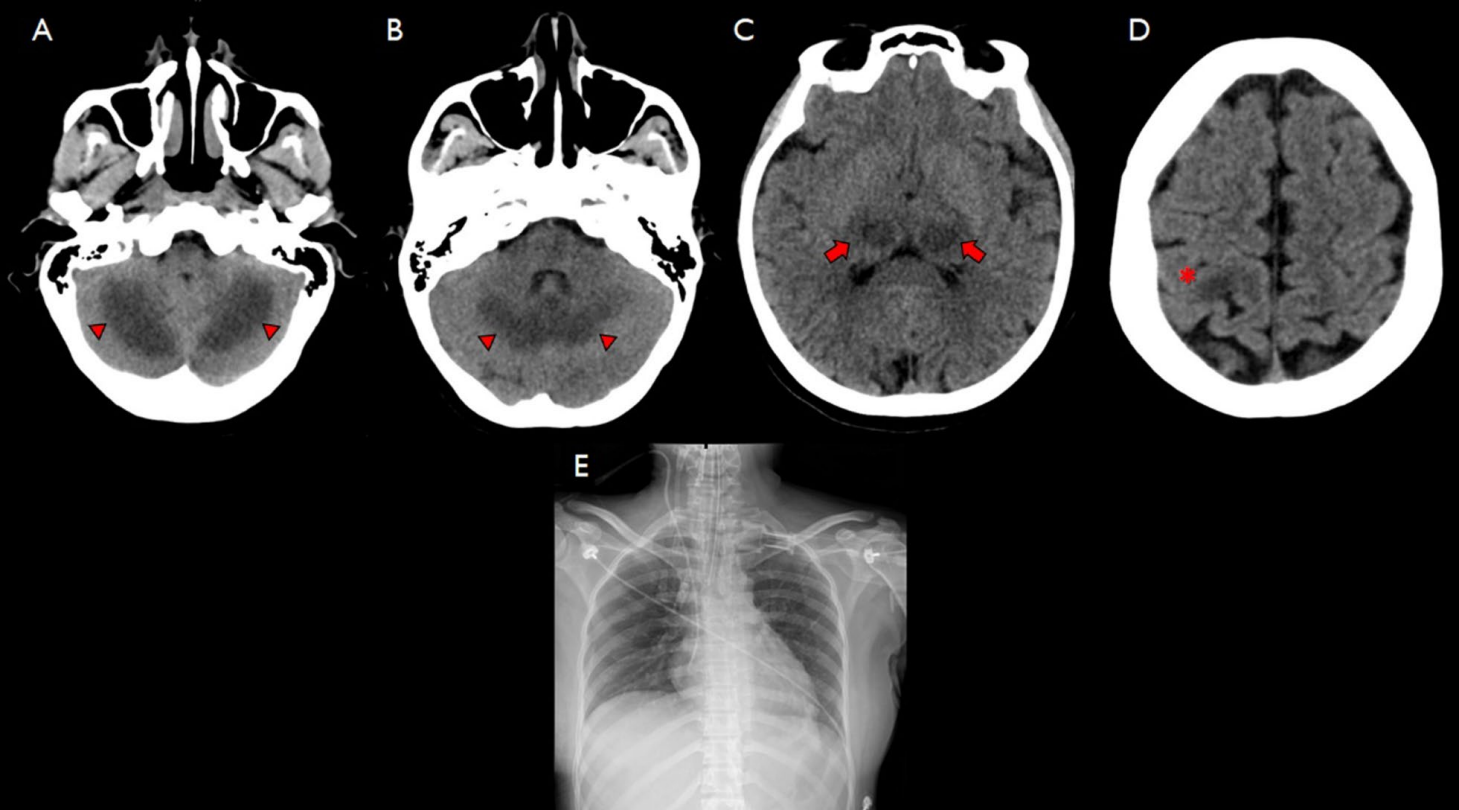
Computerized brain tomography showed bilateral symmetrical hypodense lesions involving thalami (arrows) (**C**) and cerebellar hemispheres (arrowhead), causing effacement of cerebellar folia (**A**, B); isolated hypodense lesion at left high frontal area (asterixis) (**D**). Chest X-ray after intubation showed endotracheal tube tip 0.7 cm above carina, parenchyma was normal with no obvious infiltration (**E**)

Upon arrival, the patient received intubation, fluid resuscitation, a vasopressor along with levetiracetam to control myoclonus. Non-contrast computerized tomography (CT) of the brain showed symmetrical hypodense lesions involving bilateral thalami (Fig. 1C) and bilateral cerebellar hemispheres (Fig. 1A, B), causing effacement of cerebellar folia. Another isolated hypodense lesion was observed at the left high frontal area (Fig. 1D).

The initial blood count revealed normal red blood cell count, normal white blood cell count with neutrophil predominance, thrombocytopenia (122,000 cell/uL), and coagulopathy; INR 2·44; aPTT 75·1/24·4 s; and low fibrinogen 1·34 g/L. d-dimer was markedly elevated (> 10,000 ng/mL). The initial blood chemistry showed acute kidney injury and elevated liver enzymes. In addition, markedly elevated inflammatory biomarkers were observed; serum IL-6 7390 pg/mL; ferritin 14,752 ng/mL; procalcitonin 63.05 ng/mL; and high sensitivity C-reactive protein 89·68. Comprehensive 48 cytokines panel was obtained on the fourth day of admission (Table 1).

**Table 1:** Human cytokines and chemokines panel, 48-plex (all units in pg/mL)

FGF basic = 103.5	IL-2 = 76	IL-10 = 85	MIP-1α = 1928.5
Eotaxin = 1362	IL-4 = 159	IL-12 (p70) = 22	MIP-1β = 3127.5
G-CSF = 592.5	IL-5 = 73	IL-13 = 18	PDGF-BB = 284
GM-CSF = 37	IL-6 = 2011	IL-15 = 51.5	RANTES = 4791.5
IFN-γ = 234	IL-7 = 20	IL-17A = 58	TNF-α = 282.5
IL-1β = 87	IL-8 = 756	IP-10 = 9696	VEGF = 85
IL-1ra = 840.5	IL-9 = 160	MCP-1(MCAF) = 270	CTACK = 2746
IL-1α = 77.5	GRO-α = 186	MIG = 9626	MIF = 1020
IL-2Rα = 1179.5	HGF = 1201	β-NGF = 76	TRAIL = 118.5
IL-3 = 51	IFN-α2 = 57	SCF = 1514	IL-18 = 1268.5
IL-12 (p40) = 35	LIF = 79	SCGF-β = 2422	M-CSF = 979.5
IL-16 = 635.5	MCP-3 = 152	SDF-1α = 502	TNF-β = 481

Urinalysis found pyuria and bacteriuria. Urine culture was positive for *Escherichia coli*. Stool culture and hemoculture were unremarkable. SARS-CoV-2 was detected by PCR from nasopharyngeal and throat swab and was later confirmed by whole genome sequencing to be the Delta variant. *RANBP2* gene analysis was sent out to search for a possible explanation of the cause of ANE and found the heterozygous Thr585Met mutation.

Chest radiography revealed no infiltration (Fig. 1E). Non-contrast CT of the whole abdomen found A 0·6 × 0·8 cm left distal ureteric stone situating just above the left ureterovesicular junction, causing upstream dilatation.

The final diagnoses were COVID-19, ANE, acute cerebral infarction, septic shock from urinary tract infection (UTI), and multiorgan failure. Remdesivir 100 mg intravenous (IV) once daily and dexamethasone 6 mg IV once daily were given as well as ceftriaxone 2 g IV once daily. Urinary diversion was not performed.

On the second day of admission, myoclonus was controlled but the patient deteriorated into comatose state. Blood tests revealed further worsening of renal and liver status. She was palliated due to multiorgan failure with no response to treatment. Upon the patient’s relatives will, invasive investigations including further extensive blood tests including the neutralizing antibodies against SARS-CoV-2 and serum IgG against spike proteins, as well as lumbar puncture were not done as it would be unlikely to affect management and due to the limited resource situation in Thailand during the delta outbreak. She passed away on the sixth day of admission.

## Discussion and conclusions

SARS-CoV-2 infection has caused a various range of serious neurologic complications including strokes, seizures, autoimmune diseases, and encephalopathies via several mechanisms comprising direct invasion, immune dysregulation, hypoxemia, thrombosis, multiorgan dysfunction, and cytokine storm [4]. ANE, one of the serious encephalopathies of the brain, is believed to be caused by cytokine storm following viral infection rather than direct viral invasion itself [2]. Other causes of ANE have been described in the literature; however, there has been limited solid evidence. Bacterial infection is an extremely rare cause of ANE. Only one case report described a patient who had *Escherichia coli* and *Neisseria gonorrhoeae* urinary tract infection and subsequently developed ANE [5]. Another possible cause is vaccination; however, there are only few reports that ANE developed after recent vaccination. One case reported a previously healthy 6-month-old boy who was admitted to hospital with lethargy, hypotonia and focal clonic seizures 6 days following diphtheria, tetanus toxoid and whole-cell pertussis vaccination. ANE was diagnosed based on an exclusion of possible diseases accompanied with clinical and laboratory examinations, and imaging findings [6]. Another case reported a 75-year-old Caucasian female who presented to the hospital with altered level of consciousness and dysarthria. She was diagnosed with ChAdOx1nCoV-1 vaccine associated ANE. She received her first dose of the ChAdOx1nCoV-1 vaccine 2 days prior to hospitalization and was also on a course of cephalexin for a presumed UTI. Testing of the patient’s serum for SARS-CoV-2 antibodies was negative [7]. Apart from cytokine storm, gene mutation has also been reported to precipitate ANE [2].

As mentioned above, two main causes that induced ANE includes over production of cytokine and gene mutation. For cytokine production, IL-6 and TNF-alpha, at high levels can be neurotoxic and are the main cytokines causing proteolytic destruction of blood brain barrier. This process leads to both cytotoxic and vasogenic edema followed by petechial hemorrhages and necrosis [2]. *RANBP2* encodes a nuclear pore protein that mainly functions throughout the cell cycle, its mutations are strongly associated with ANE via multiple processes including mitochondrial function, viral entry, cytokine signaling, immune response, and blood–brain barrier maintenance [2]. The *RANBP2* mutation also causes cytokine storm, blood brain barrier disruption, impaired mitochondrial trafficking, and increased vulnerability to oxidative damage in setting of infection [8]. A previous study in Japan showed that high serum IL-6 (> 15,000 pg/mL) is an indicator of a very poor prognosis in ANE in children (100% mortality) [3]. Since the emergence of COVID-19, *RANBP2* gene mutation has never been reported in COVID-19 cases. Therefore, this is the first case report. We believe that *RANBP2* gene plays a crucial role in the propagation of ANE and might support the poor outcome and high IL-6 level in our patient. Furthermore, most of the COVID-19 related ANE patients from literature review [9] had severe pulmonary involvement, which needed intubation and invasive respiratory support; although, our case did not have pulmonary involvement, which is a unique case. We proposed that the extremely high IL-6 level might be responsible for the formation of ANE without the involvement of the respiratory symptoms. This has been reported in 6-year-old-boy of influenza virus associated ANE without pulmonary involvement in Thailand which resembles what we found in our case [10].

Hyper-responsiveness of the immune response and autoinflammatory phenomenon in patients with COVID-19 have been increasingly reported [11]. IL-6 has been considered to be a promising marker of COVID-19 severity in which the level of IL-6 was found higher in severe cases compared with mild cases [12]. Thus, it is believed to be deemed as a prognostic factor [13, 14] and a potential treatment target [15]. However, the exact cut-off level of IL-6 regarding poor prognosis is unknown. One study found that mean level was only 21.55 pg/mL in severe COVID-19 (ranging from 1.5 to > 5000 pg/mL). From the study mentioned, it could be interpreted that the majority of patients did not have a high IL-6 level, despite the critical illness of the disease [16]. However, it should be noted that acute elevated pro-inflammatory markers in COVID-19 are followed by immune suppression. In other words, the elevated level might be due to the timing of the blood collection [17]. Although the IL-6 level in our case might be in the acute phase, the uniqueness of an extremely high IL-6 level could be precipitated from other unmentioned causes. Therefore, leading to a search for further contribution to this increased IL-6 level.

Vaccination is crucial for stopping the outbreak by raising humoral and cell-mediated immunity [18]. However, antibody-dependent enhancement (ADE) exacerbating the severity of COVID-19 has been of great concern. The term ADE was first proposed in respiratory syncytial virus (RSV) and measles in which antibodies increase the severity of pathogens. The antibody-based vaccines including inactivated viral vaccines, which may contain non-neutralizing antigen targets and/or the S protein in non-neutralizing conformations, has a major concern of providing a non-protective target for antibodies that could increase the inflammation via ADE mechanisms like other respiratory pathogens such as RSV, Dengue virus, Zika virus, and Middle East Respiratory Syndrome Coronavirus [19]. Two proposed mechanisms are enhancement of antibody-mediated virus uptake into Fc gamma receptor IIa (FcγRIIa) of the phagocytic cells and via the immune complex formation [19]. In COVID-19, it is believed to involve both mechanisms. Antibodies that are responsible for the formation of immune complexes are non-neutralizing antibodies. They are antibodies that could bind to the virus but could not neutralized them. The concept of ADE mechanism of SARS-CoV-2 pseudoviral infection in vitro has recently proved that FcγRIIB-mediated uptake of SARS-CoV-2/mAb complex with bivalent. They reported that two neutralizing mAbs, MW01 and MW05, could enhance the infection of SARS-CoV-2 pseudovirus on FcγRIIB expressing B cells, which might lead to an increase in severity of viral infection [20]. Therefore, forming a complement cascade. In conjunction with previous findings, it has been documented in RSV that non-neutralizing antibodies bind with viral antigens to initiate a complement cascade, secrete pro-inflammatory cytokines and recruit immune cells; later creating an over production of cytokine [21]. Not all vaccines are reported to be responsible for the formation of immune complexes. Newer generations of vaccines (mRNA vaccines) that could produce S-specific neutralizing antibodies have a lower risk of ADE [22]. On the contrary, inactivated vaccines elicit the non-neutralizing S protein which is hypothesized to possibly drive the production of inflammation [19].

The Brighton Collaboration Vaccine-associated Enhanced Disease Working Group has proposed the definition of the term “Vaccine Associated Enhanced Disease (VAED)” published in Vaccine, 2021 [23]. Regarding the best available information in this case, the level of certainty of VAED could be a possible case of VAED (level 3B). We lack the immunopathology in target organs involved by histopathology such as presence of tissue eosinophils, elevated pro-inflammatory Th2 cytokine in tissue, complement activation, etc. Moreover, we differentiate vaccine failure, defined as the occurrence of the specific vaccine-preventable disease in a person who is appropriately and fully vaccinated, from VAED. In our case, the patient developed symptoms after 8 days after her 1^st^ dose of an IVV, BBIBP-CorV; thus, the effective protective response for SARS-CoV2 infection might not have reached its maximum efficacy. According to the originally published phases 1 & 2 studies, more than 75% of vaccine recipients in the group aged 18–59 years seroconverted after the first vaccine dose (day 14) [24]. The remaining vaccine recipients seroconverted on day 28 and 100% seroconversion was found in all participants on day 42. The second booster dose immunization raised the significantly greater neutralizing antibody titers than the days 0 and 14 schedule and the single immunization [24]. Therefore, vaccine failure should also be taken into consideration in this case.

In regard to our patient’s clinical history, physical examination, laboratory diagnosis, genetic studies, and imaging studies, we could establish the diagnosis of ANE based on the proposed diagnostic criteria [2] by Mizuguchi et al. [25] and Neilson et al. [26]. We believe that SARS-CoV2 infection, *RANBP2* gene mutation, bacterial infection, and recent inactivated vaccination are separate events that interact with each other to cause over production of cytokines. Therefore, we hypothesized that all the above mentioned contributes to the formation of ANE in different degrees.

Further study is needed to confirm this hypothesis, specifically in the post-vaccination period. It is crucial that IVV should be carefully monitored and observed for safety concerns related to its possible complications. In addition, Role of *RANBP2* mutation and its application in COVID-19 and ANE should be further elaborated.

We present an extremely rare case of ANE in COVID-19 with extremely high interleukin-6 and RANBP2 mutation despite the absence of significant pulmonary involvement. The combination of SARS-CoV2 infection and bacterial infection (culture proven) accompanying with predisposing genetic factor, *RANBP2* gene mutation were the possible main causes of the illness. In addition, non-neutralizing antibodies caused by the 1^st^ dose of IVV might play a possible role via ADE mechanism. Further study is needed to confirm this hypothesis, specifically in the post-vaccination period. We believe that multiple etiologies in our case precipitated the propagation of this condition and requires further attention for future studies.

## Data Availability

All data and materials are available with the first author (TP) and corresponding author (WR). Whole genome sequencings are available via The Global Initiative for Sharing All Influenza Data (GISAID) database and can be assessed by registered GISAID users using the accession number EPI_ISL_5937717.

## Abbreviations

ADE: Antibody-dependent enhancement
ANE: Acute necrotizing encephalopathy
CT: Computerized tomography
COVID-19: Coronavirus disease-2019
FcγRIIa: Fc gamma receptor IIa
IL: Interleukin
IV: Intravenous
IVV: Inactivated virus vaccine
RANBP2: Ran Binding Protein 2
RSV: Respiratory syncytial virus
SARS-CoV-2: Severe acute respiratory syndrome coronavirus 2
TNF: Tumor necrosis factor
Th: T helper lymphocyte
VAED: Vaccine associated enhanced disease
